# Identification, Expression, and Functions of the Somatostatin Gene Family in Spotted Scat (*Scatophagus argus*)

**DOI:** 10.3390/genes11020194

**Published:** 2020-02-12

**Authors:** Peizhe Feng, Changxu Tian, Xinghua Lin, Dongneng Jiang, Hongjuan Shi, Huapu Chen, Siping Deng, Chunhua Zhu, Guangli Li

**Affiliations:** 1 Fisheries College, Guangdong Ocean University, Zhanjiang 524088, China; Fengpz@163.com (P.F.); tiancx@gdou.edu.cn (C.T.); lxh_13414934257@163.com (X.L.); jdn1987@163.com (D.J.); shihongjuan1990@163.com (H.S.); chpsysu@hotmail.com (H.C.); dengsp@gdou.edu.cn (S.D.); zhu860025@163.com (C.Z.); 2 Guangdong Research Center on Reproductive Control and Breeding Technology of Indigenous Valuable Fish Species, Zhanjiang 524088, China; 3 Marine Ecology and Aquaculture Environment of Zhanjiang, Zhanjiang 524088, China

**Keywords:** *Scatophagus argus*, somatostatin family, tissues expression, vitro incubation

## Abstract

Somatostatins (SSTs) are a family of proteins consisting of structurally diverse polypeptides that play important roles in the growth regulation in vertebrates. In the present study, four somatostatin genes (*SST1*, *SST3*, *SST5*, and *SST6*) were identified and characterized in the spotted scat (*Scatophagus argus*). The open reading frames (ORFs) of *SST1*, *SST3*, *SST5*, and *SST6* cDNA consist of 372, 384, 321, and 333 bp, respectively, and encode proteins of 123, 127, 106, and 110 amino acids, respectively. Amino acid sequence alignments indicated that all SST genes contained conserved somatostatin signature motifs. Real-time PCR analysis showed that the SST genes were expressed in a tissue specific manner. When liver fragments were cultured in vitro with synthetic peptides (SST1, SST2, or SST6 at 1 μM or 10 μM) for 3 h or 6 h, the expression of insulin-like growth factor 1 and 2 (*Igf-1* and *Igf-2*) in the liver decreased significantly. Treatment with SST5 had no significant effect on *Igf-1* and *Igf-2* gene expression. This study provides an enhanced understanding of the gene structure and expression patterns of the SST gene family in *S. argus*. Furthermore, this study provides a foundation for future exploration into the role of SST genes in growth and development.

## 1. Introduction

Somatostatins (SSTs) are cyclic regulatory peptides that play an important role in reproduction, metabolism, and the growth of vertebrates [1]. The expression of SST peptides is predominantly localized to the central nervous system and peripheral nerves, and secondarily in the intestine, stomach, and pancreas. These proteins function as both a hormone and a neurotransmitter [2]. First isolated from an ovine hypothalamus extract by Brazeau in 1973, the SS-14 amino acid was observed to inhibit the secretion of growth hormone (*Gh*) from the pituitary of rats [3]. It has been shown that SST not only directly inhibits the secretion of *Gh*, but also inhibits the growth of the body through the regulation of the *Gh*-*Igf-1* system [4]. In teleost fishes, SSTs exert their effects through the regulation of *Igf-1* synthesis and secretion in a tissue dependent manner. For example, in salmon (*Salmo salar*), secretion of SS-14 from the hypothalamus can inhibit the expression of *Igf-1* in the liver [5,6]. In rainbow trout (*Oncorhynchus mykiss*), the expression of *Igf-1* in plasma and liver was reduced by injection of SS-14 [6], and similarly inhibited *Igf-1* in vitro in hepatocyte cultures [7]. However, the repertoire of SST genes in the spotted scat (*Scatophagus argus*), as well as the locations of their expression and the functions of the mature peptides, have not yet been studied.

It has been reported that different forms of SST in teleost species are the products of paralogous genes of at least six ancestral SST genes named *SST1*, *SST2*, *SST3*, *SST4*, *SST5,* and *SST6*. In vertebrate species, the SST gene family has been found to be widely distributed, being reported in cartilaginous fishes, ray-finned fishes, birds, reptiles, and amphibians [1,2,3]. The *SST1* and *SST2* genes likely arose through the 1R/2R whole genome duplications early in vertebrate evolution. The *SST1* gene has been observed in both fish and mammals [3,8]. The *SST2* gene, also known as *cortistatin* (*CORT*) in mammals, is expressed in many vertebrates such as human (*Homo sapiens*) [9,10], chicken (*Gallus gallus*) [11], and African lungfish (*Protopterus annectens*) [12]. In grouper (*Epinephelus coioides*) [13] and common carp (*Cyprinus carpio*) [14], three *SST* genes (*SST1, SST3,* and *SST6*) have been observed. In dogfish (*Scylorhinus canicula*) [15] and lamprey (*Lampetra japonicum*) [16], five *SST* genes (*SST1, SST2, SST3, SST5,* and *SST6*) and three *SST* genes (*SSTa, SSTb,* and *SSTc*) have been observed, respectively, although the phylogenetic relationship of the three lamprey SST genes remains unclear. Two forms of SST (*SST1* and *SST2*) have been found in tetrapods [10,17,18,19]. In contrast, *SST3* and *SST6* are known in actinopterygians, *SST4* in teleosts, and *SST5* in Chondrichthyes, Actinopterygians, and Actinistia [20,21,22,23].

It has been proposed that the SST gene family was shaped by the two rounds of genome-wide duplication (1R and 2R) and the teleost fish-specific genome doubling (3R) [24,25,26,27]. *SST1*, *SST2,* and *SST5* arose in 1R/2R and existed in the common ancestor of extant vertebrates. [21]. Subsequently, the *SST4* gene arose during the tetraploidization (3R) of teleost fishes, approximately 350 million years ago, while the *SST3* and *SST6* genes were generated by tandem duplication of the *SST1* and *SST2* genes during the same period [28]. It is believed that the *SST5* gene was lost in tetrapods [20], and the *SST4* gene was thought to be lost in *Scylorhinus canicula* [27]. There seems to have been several differential losses of SST genes in different vertebrate lineages. Therefore, it is worth investigating whether the spotted scat has the full repertoire of genes present in other teleost fishes.

The spotted scat (*S. argus),* which belongs to the Perciformes, is an economically important species of marine fish for both the ornamental pet trade and human consumption. The species is mainly distributed throughout the Indian and Pacific Oceans [29]. Only one subspecies of *S. argus* can be found in the waters along the coast of China, and is restricted to the northern part of the South China Sea and the southern part of the East China Sea [30,31]. This species is rich in nutrients and has broad appeal as a food source in China. The genome and transcriptome of *S. argus* were recently sequenced (unpublished data), which set the stage for the analysis of the SST gene family reported here.

In the present study, four members of the SST gene family (*SST1*, *SST3*, *SST5*, and *SST6*) were identified in *S. argus* by phylogenetic and synteny analyses from representative vertebrate species and *S. argus* genome sequences (unpublished data, see Methods below). Then, the tissue differential expression of each SST gene was determined by qRT-PCR. Finally, liver tissue fragments were incubated in vitro with different concentrations of SSTs, and expression of *Igf-1* and *Igf-2* were measured at 3 and 6 h, respectively. This study improves the current understanding of the function of the SST gene family in teleost fishes, and establishes a foundation for future exploration of the growth and developmental mechanisms of *S. argus*. The nomenclature of the genes in the present study is based on the convention as proposed by Tostivint et al. (2019) [27].

## 2. Materials and Methods

### 2.1. Cloning of the Somatostatin Genes of S. argus

A total of 20 *S. argus* (150–200 g) were acquired from Donghai Island, Zhanjiang, Guangdong Province, China. All animal experiments were carried out in accordance with the guidelines and approval of the Animal Research and Ethics Committee of the Institute of Aquatic Economic Animals, Guangdong Ocean University (201903004). All fish were anesthetized with 100 mg/L of tricaine methane sulfonate (MS 222, Sigma, Saint Louis, MO, USA) and dissected. Total RNA was extracted from each pooled tissue sample. First strand cDNA synthesis was performed using the PrimeScript™ RT Reagent Kit (Takara, Otsu, Japan) in conjunction with the gDNA Eraser Kit, according to the manufacturer’s instructions. From each sample, 1 µg of RNA was set aside for cDNA synthesis, and stored at −20 °C until use. Based on published SST nucleotide sequences, four pairs of primers (Table S1) were designed to amplify the Open Reading Frames (ORFs) of the four SST genes. Next, Polymerase Chain Reaction (PCR) amplification was performed using 2 × PCR MIX (Dongsheng Biotechnology Co. Ltd., Guangzhou, China). The thermocycling protocol used was as follows: 94 °C (30 S); 58 °C (30 S), and 72 °C (30 S) for 35 cycles, followed by a final extension at 72 °C for 10 min. The amplified fragments were resolved by electrophoresis on 1.5% agarose gels. The target fragment was purified using a DNA Gel Extraction Kit (Guangzhou Dongsheng Biotech-Specializes in Molecular Biology, Guangzhou, China). The purified fragments were subsequently cloned into the pEASY-T3 (TransGen Biotech, China) plasmid for verification by sequencing (Sangon Biotech, Shanghai, China).

### 2.2. Identification of SSTs from Representative Vertebrate Species

The SST sequences of mandarin fish (*Siniperca chuatsi*) and zebrafish (*Danio rerio*) were downloaded from the National Center for Biotechnology Information (NCBI) database. Based on these SST sequences, four homologous genes were identified in the *S. argus* genomes and transcriptomes here using a local blast program [32] (Figure S3). Female adult spotted scats, reared in Zhanjiang Donghai Island Cultivation Base (Zhanjiang, Guangdong, China) were used for genome sequencing and assembly. The extracted muscle DNA molecules were sequenced with both Illumina HiSeq X Ten platform (Illumina Inc., San Diego, CA, USA) and PacBio Sequel platforms (Pacific Biosciences of California, Menlo Park, CA, USA). Using PacBio sequencing and the Hi-C technique to assemble the *S. argus* genome, the genome scale was 572.42 Mb with 240 contigs and contig N50 length of 19.60 Mb. A total of 187 contigs were reliably assembled into 24 chromosomes, representing 99.73% of the total genome. Among the 4584 total Benchmarking Universal Single Copy Orthologs (BUSCO) groups searched, more than 98.28% of the BUSCO genes were successfully detected in the genome. Specific primers were designed to verify the SST gene sequences in the genome and transcriptome by PCR amplification and cloning of genes. Finally, these cloned sequences (instead of predictions from the genome) were used for subsequent analysis. The amino acid, CDS (coding sequence) length, and number of chromosomal loci of the SST genes were noted based on the genomic database. The theoretical molecular weight (kDa) and pI (isoelectric points) of all identified proteins were calculated using the Lasergene v7.1.0 software.

The ORFs of gene orthologs in additional vertebrates, namely zebrafish (*D. rerio*), fugu (*Takifugu rubripes*), medaka (*Oryzias latipes*), tilapia (*Oreochromis niloticus*), coelacanth (*Larimichthys crocea*), stickleback (*Gasterosteus aculeatus*), Chinese sturgeon (*Acipenser sinensis*), African lungfish (*P. annectens*), mandarin fish (*S. chuatsi*), grouper (*Epinephelus coioides*), channel catfish (*Ictalurus punctatus*), dogfish (*S. canicula*), elephant shark (*Callorhinchus milii*), rainbow trout (*O.rhynchus mykiss*), tetraodon (*Tetraodon nigroviridis*), gold fish (*Carassius auratus*), white sucker (*Catostomus commersonii*), platyfish (*Xiphophorus maculatus*), fathead minnow (*Pimephales promelas*), American anglerfish (*Lophius americanus*), red-bellied piranha (*Pygocentrus nattereri*), frog (*Xenopus laevis*), lizard (*Anolis carolinensis*), chicken (*G. gallus*), human (*H. sapiens*), mouse (*Mus musculus*), and crab-eating macaque (*Macaca fascicularis*) were collected from the NCBI and Ensembl databases. Next, the translation program in ExPASy [33] was used to predict the amino acid sequences of *S. argus* SSTs. Signal peptide sequences and transmembrane domains were predicted online using the SignalP-5.0 Server [34]. Amino acid sequence identity comparisons were performed using the Clustal W algorithm in the Lasergene v7.1.0 software. The CDS of four SST genes were blasted with the genomic data to obtain the structure of introns and exons. In addition, a Simple Module Architecture Research Tool (SMART) [35] was used to identify the protein structures based on sequence homology.

### 2.3. Phylogenetic Analysis and Amino Acid Alignment

Multiple alignments of the analyzed SST protein sequences were performed using the Clustal W algorithm in the Lasergene v7.1.0 software. A phylogenetic tree was constructed using the maximum likelihood (ML) method using the Mega 7.0 software with the JTT + F model of substitution combined with the neighbor-joining interchange (NNI) method. The degree of confidence for each branch point was determined by bootstrap analysis (500 replicates).

### 2.4. Syntenic Analyses

In order to produce synteny maps of the SST genes in *S. argus*, syntenic analyses were performed by examining the conserved colocalization of adjacent genes in the reference genomic sequence of *S. argus* and the genomes of other selected species. Genes flanking the SST genes in other species (except *S. argus*) were identified from the Ensembl [36] and NCBI [37] databases. Genes surrounding each zebrafish SST gene were used to search for orthologs in the *S. argus* genome database by local BLAST and BLASTP. Non-protein-coding genes and genes encoding unknown proteins were excluded from further analysis.

### 2.5. Tissue Specific Analysis of SST Gene Expression by qRT-PCR

Total RNA was extracted from 11 tissues of each *S. argus* (hypothalamus, pituitary gland, gonad, liver, spleen, kidney, heart, muscle, gill, intestine, and stomach). In order to accurately verify the tissue distribution of different genes, qRT-PCR was employed. New specific primers were designed, and the expression of the corresponding genes was detected (Table S1). The reactions were performed using an ABI 7500 real-time theromcycler. A SYBR Green Real time PCR Master Mix (Toyobo, Japan) was used according to the manufacturer’s protocol. The thermocycling profile used was as follows: denaturation at 94 °C for 5 min, followed by 40 cycles at 94 °C for 15 s, 58 °C for 15 s, and 72 °C for 20 s. The 2^ΔΔCt^ method was applied to calculate the relative expression of different genes in different tissues, with *β-actin* being used as the internal reference gene.

### 2.6. Expression of Igf-1 and Igf-2 in the Liver After Injection with SSTs

Liver tissue samples were cultured as previously described by Wang et al. [38]. Briefly, livers were carefully dissected, placed in a dish containing 2 mL of culture medium, and washed three times with M199 medium. The tissue was then cut into small pieces, and transferred into a 24-well culture plate. Each well contained 2 mL of M199 medium supplemented with penicillin (100 IU/mL) and streptomycin (100 μg/mL). Synthetic SST polypeptides were prepared (SST1-amide, AGCKNFFWKTFTSC-amide; SST3-amide, APCKNFFWKTFTSC-amide; SST5-amide, AGCRNFFWKTFTSC-amide; SST6-amide, AGCKNFYWKGFTSC-amide) from GL Biochem Ltd. (Shanghai, China). Next, SSTs were dissolved in M199 (Mediatech, Manassas, VA) at a stock concentration of 100 μM. After pre-incubation at 25 °C for 2 h in a 5% CO_2_ humidified incubator, the medium was replaced with fresh media containing the SST. Three concentrations of synthetic SST were tested (0.1, 1, and 10 μM) at one of two different incubation times (3 or 6 h), together with a control group. Each condition consisted of three replicates and this was repeated in triplicate. After incubation for 3 or 6 h, each liver sample was collected and stored at −80 °C for analysis of gene expression by qRT-PCR.

### 2.7. Statistical Analysis

Data were presented as the mean ± S.E.M. Significant differences in the data among groups were estimated using one-way analysis of variance (ANOVA) followed by Duncan’s post-hoc test using SPSS 18 software. Differences were considered to be statistically significant at *p* < 0.05.

## 3. Results

### 3.1. Identification of the SST Gene Family in S. argus

In the present study, four genes *SST1*, *SST3*, *SST5*, and *SST6* were successfully identified in *S. argus*. The lengths of the cDNAs of these genes were 658, 790, 748, and 752 bp, respectively (Figure S1). The lengths of the SST proteins ranged from 101 to 127 amino acids, with putative molecular weights ranging from 11.59 to 14.19 kDa. The theoretical isoelectric points (pI) for these proteins were predicted to be between 5.14 and 9.06 (Table 1). Both *SST1* and *SST3* were located on linkage group 4 (Table 1), whereas *SST5* and *SST6* were located on linkage group 23 and linkage group 8, respectively. The SST1 sequence contained an arginine-lysine (R-K) and an arginine (R) recognition site, which are predicted to produce a 14-aa peptide somatostatin (SS-14) and a 30-aa peptide somatostatin (SS-30), respectively. The SST3 sequence included one arginine-lysine (R-K) and one arginine (R) recognition sites, which are predicted to produce 14- and 28-aa peptide somatostatins (SS-14 and SS-28). The SST5 sequence included an arginine-lysine (R-K) and an arginine (R) recognition site, which are predicted to produce 14- and 24-aa peptide somatostatins (SS-14 and SS-24), respectively. The SST6 sequence included an arginine-lysine (R-K) and an arginine (R) recognition site, potentially yielding mature 14- and 23-aa peptide somatostatins (SS-14 and SS-23) (Figure S1).

### 3.2. Gene Structures and Conserved Domains

**Figure 1:**
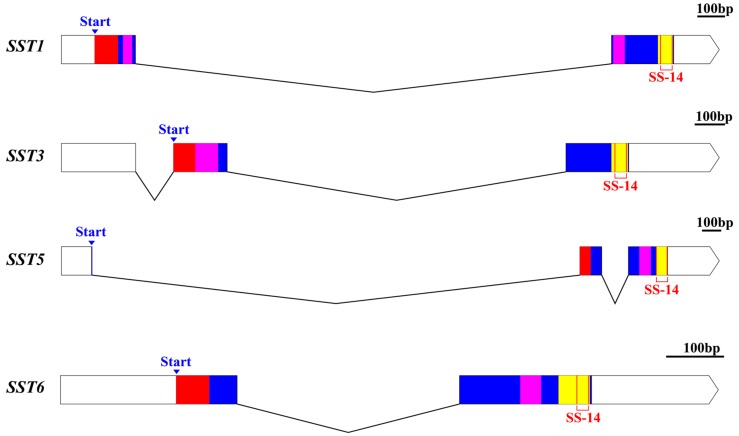
Schematic diagram of the exon–intron structure and protein domain prediction of SST genes in *S. argus*. The white rectangle on the left represents the 5’UTR and the white polygon on the right depicts the 3’UTR. The open reading frame is indicated by colored boxes. The blue arrows represent the start codon position, and signal peptides are marked in red, the low complexity and Pfam: Somatostatin regions are marked in purple and yellow, respectively. Regions encoding mature peptides are indicated by orange boxes. The short solid lines at the top right are scale bars, representing 100 bp.

The protein structures of the SST gene family were predicted using SMART (Figure 1). Both the *SST1* and *SST6* genes of *S. argus* contained a single intron and two exons. Both the *SST3* and *SST5* genes contained two introns and three exons (Figure 1). It was observed that each of the four SST genes contained a conserved domain (Pfam: Somatostatin region especially SS-14 amino acid), throughout their genetic evolution (Figure S2). The SS-14 amino acid sequence of the SST genes were highly conserved among different species. However, some domains were divergent between species. For example, the SST1 protein contained two regions of low-complexity, whereas all others examined contained only one low complexity region.

### 3.3. Phylogenetic and Syntenic Analyses of S. argus SST Genes

**Figure 2:**
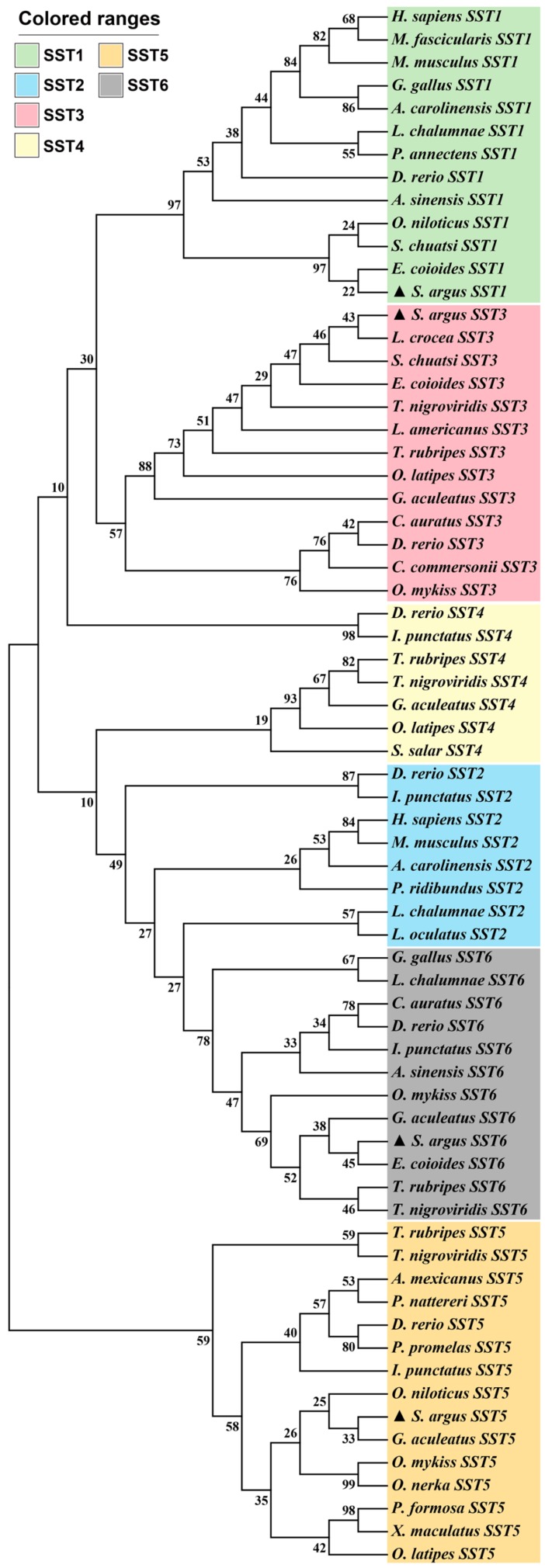
Phylogenetic tree of the SST family members in selected vertebrate species. The tree was constructed using mega 7.0 and the maximum likelihood approach. Bootstrap testing was based on 500 replicates. The SST genes of *S. argus* are denoted by triangles. Genbank accession numbers of these genes are provided in Table S2.

**Figure 3:**
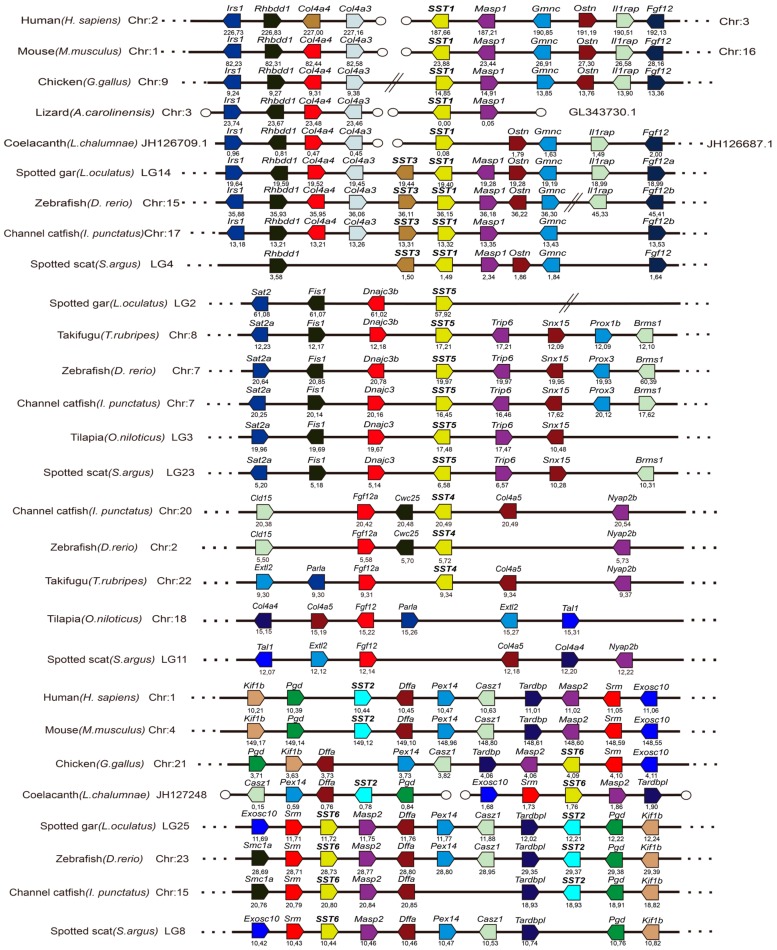
Conserved synteny analysis of SST genes from *S. argus* using the genomes of 10 selected vertebrate species (human, mouse, chicken, lizard, coelacanth, spotted gar, zebrafish, channel catfish, tilapia, and fugu). Genes are represented by pentagons, and the directions of the reading frames are represented by the direction that the pentagon is pointing. Empty circles indicate the end of scaffolds. Gene positions (in megabases -Mb) are displayed below each pentagon. The detailed chromosomal positions of the genes used are presented in Table S3.

In order to analyze the evolutionary relationships of SST1, SST3, SST5, and SST6 in *S. argus* with other species, a phylogenetic tree based on the amino acid sequences was constructed (Figure 2). It was observed that the four SST genes from *S. argus* clustered into four distinct branches, along with orthologous genes of other vertebrate species, although the support for the SST3, SST5, and SST6 branches was low. Additionally, *S. argus* was most closely related to the scorpion fish. The SSTs exhibited high amino acid identity with other vertebrate species analyzed here. Specifically, SST1, SST3, SST5, and SST6 shared 45.2–93.5%, 47.4–92.1%, 40.2–77.9%, and 52.7–96.1% similarity to other known vertebrate SSTs (Table S2), respectively. The SST1 and SST3 amino acid sequences shared the highest sequence identities with their grouper (*E. coioides*) orthologs, which were 93.5% and 92.1%, respectively. The SST5 and SST6 shared the highest sequence identities with tilapia (*O. niloticus*) and grouper (*E. coioides*), which were 77.9% and 96.1%, respectively. For these four SST genes, syntenic analyses were carried out to lend additional support to the accuracy of the SST phylogenetic analysis. Conserved syntenic regions were identified, and the SST orthologs from *S. argus* and other species were determined. Taken together, these analyses suggest that the annotations attributed here were, in fact, appropriate (Figure 3).

### 3.4. Tissue Specific Distribution of SST Gene Expression

**Figure 4:**
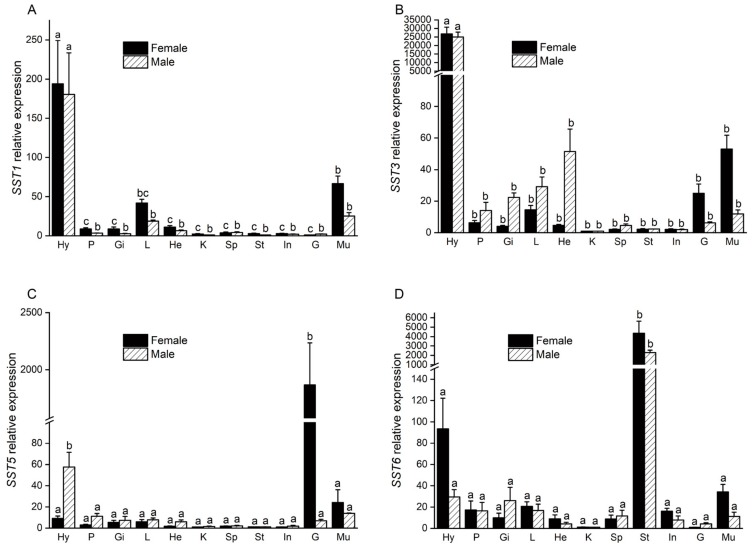
The relative expression levels of female and male *S. argus SST1* (**A**), *SST3* (**B**), *SST5* (**C**), and *SST6* (**D**) in different tissues. The error bars represent the standard error of the means of three independent replicates. Significant differences between male and female fish were compared separately. Different letters above the error bar indicate statistical differences at *P < 0.05*, as determined by one-way Analysis of Variance (ANOVA) followed by a Duncan’s post hoc test. Hy: Hypothalamus; P: Pituitary; Gi: Gill; L: Liver; He: Heart; K: Kidney; Sp: Spleen; St: Stomach; In: Intestine; G: Gonad; Mu: Muscle.

The mRNA expression levels of *SST*s in different tissues were examined by qRT-PCR (Figure 4). It was observed that all four of the SSTs examined were abundantly expressed in the hypothalamus. However, specific genes were expressed differentially in a tissue dependent manner. For example, *SST1* and *SST3* were equally expressed in the liver and muscle, but the expression of *SST3* in the hypothalamus was significantly higher than that of *SST1*. Furthermore, *SST3* was expressed at modest levels in the ovaries and muscle of female fish, and modest expression was observed in the heart, liver, muscle, and other tissues of male fish. The expression of *SST5* was gender-specific, being highly expressed in the ovaries of females and hypothalamus of males. In particular, the expression level of *SST5* in the ovaries was much higher than was observed in the testis. Finally, *SST6* exhibited the highest expression in the stomach, followed by the hypothalamus and intestine.

### 3.5. Effects of SSTs on the Expression of Igf-1 and Igf-2 in Liver Samples Incubated with S. argus In Vitro

**Figure 5:**
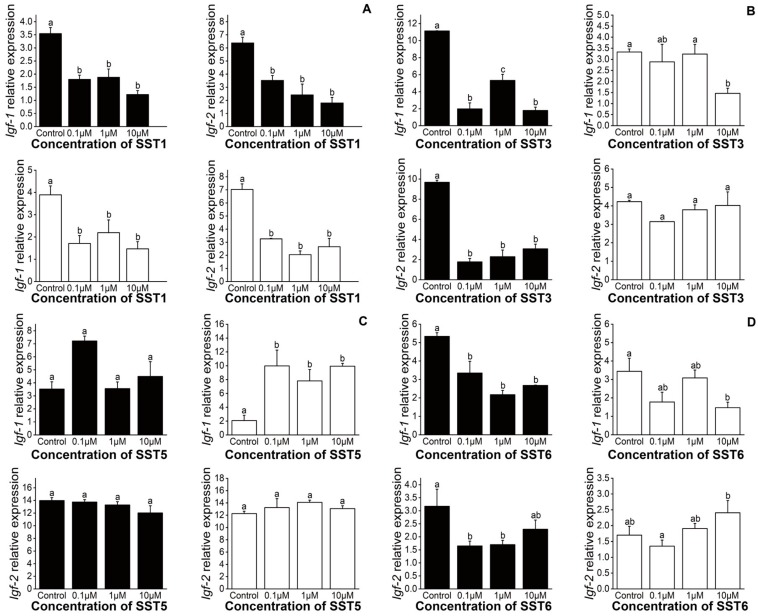
Effects of different concentrations and in vitro incubation times of SST1 (**A**), SST3 (**B**), SST5 (**C**), and SST6 (**D**) on the expression of *Igf-1* and *Igf-2* in liver. Black bars represent incubations for 3 h and incubation for 6 h in white bars. Data are presented as mean ± SEM (n = 3). Significant differences at *P < 0.05* were labeled with different letters.

Compared with the control group, SST1 significantly inhibited the expression of *Igf-1* and *Igf-2* in hepatocytes at different concentrations (control, 0.1, 1, or 10 μM) and incubation times (3 or 6 h) (Figure 5A). Similarly, SST3 inhibited *Igf-1* and *Igf-2* expression at 3 h. However, after 6 h of treatment, *Igf-1* expression was significantly decreased in the 10 μM group only (Figure 5B). At 3 h of treatment, SST5 had no discernable effect on either *Igf-1* or *Igf-2*. However, at 6 h, a significant increase in the expression of *Igf-1* was observed (Figure 5C). The expression of *Igf-1* and *Igf-2* were significantly reduced at 3 h following incubation with SST6. After 6 h, incubation of hepatocytes with 10 μM SST6 resulted in a significant decrease in *Igf-1,* and a significant increase in *Igf-2* expression (Figure 5D).

## 4. Discussion

In the present study, four members of the SST gene family (*SST1*, *SST3*, *SST5*, and *SST6*) were reported for the first time in *S. argus*. Gene structure analysis showed that *SST1* and *SST6* have two exons and one intron, *SST3* has two exons and two introns, and *SST5* has three exons and two introns. The amino acid sequences and protein domains of each SST genes were compared to those of other vertebrate species. The results showed that the mature peptides SS-14 were fully conserved in SST1 and SST6 in vertebrates (except in *Acipenser sinensis*), whereas the sequences of SST3 and SST5 varied by only 2–3 amino acids (Figure S2). These data suggest that the four SST genes are highly conserved among vertebrate species [39]. In *S. argus*, all four SST genes contain a signal peptide, a long intermediate segment, a putative cleavage site, and a C-terminal sequence [40]. However, different NH_2_ extension forms were observed in different SST genes including SS-30 residues for *SST1*; SS-28 for *SST3*, SS-24 and 28 for *SST5;* and SS-23 for *SST6*. Other types of SST peptides have similarly been observed in other teleost fish species including SS-30 in *E. coioides*, and SS-28 in *A. transmontanus* [41]. The diversity of SST peptide types in *S. argus* suggests that SST can participate in different aspects of physiological regulation and other biological functions.

Phylogenetic analysis corroborated the identity of four genes (*SST1*, *SST3*, *SST5* and *SST6*) in *S. argus* in this study. The resulting phylogenetic tree showed that the four SST genes identified were clustered into the same clade as the corresponding genes in other species. The conservation of synteny around the SST genes confirmed the identities inferred from the phylogeny. In the present study, we could determine the linkage group assignments of these genes to be linkage group 4 (*SST1* and *SST3*), 23 (*SST5*), and 8 (*SST6*), as shown in Figure 3. Previously, it was demonstrated that *SST1* and *SST3* were located on the same chromosome in zebrafish [20]. Similarly, *SST2* and *SST6* were also located on the same chromosome in teleost species. However, the *SST2* and *SST4* genes could not be identified in the *S. argus* genome in the present study. Interestingly, *SST2* has been observed in amphibians (but not xenopus) and mammals, and *SST4* (derived from *SST1*) was only found in ostariophysi [20]. The results presented here were consistent with previous conclusions, and this corroborates the hypothesis that *SST3* and *SST6* likely arose by tandem duplications from *SST1* and *SST2* in teleost fish, respectively [27]. Overall, the structures of SST genes in *S. argus* were conserved with respect to their corresponding orthologs in other teleost fishes.

Here, qRT-PCR analysis indicated that SST mRNAs were expressed differentially in the tissues examined. For example, *SST1* and *SST3* were mainly expressed in the hypothalamus, suggesting neuroregulatory functions. The fact that *SST5* was highly expressed in ovaries suggests that this gene may be involved in gonadal development and sexual dimorphism in teleost fishes. These results were consistent with those of previous studies in zebrafish [20]. Similar to reports in *E. coioides* [13], and goldfish (*C. auratus*) [42], *SST6* was highly expressed in the stomach of *S. argus*. Taken together, these observations hint at a regulatory function of the gastrointestinal tract, and may also play a role in digestion. Studies have shown that SS-14 in *Gadus morhua* can significantly reduce basal gastric acid secretion through induction of bombesin and histamine [43].

Gene duplication has been implicated as an important source of evolutionary novelty [44]. Functional differences between duplicates are necessary in order for the duplicates to be retained in the genome [45]. After duplications, the daughter genes are under loose constraint and mutations in the regulatory regions of the genes may be responsible for the observed distinct expression patterns of SST genes [46]. The results presented here further suggest that the different SST genes have different functions in teleost fishes, as evidenced by their differential expression patterns. However, more thorough studies are required to verify the functionality of each SST gene.

Somatostatin plays an important role in the growth of teleost fishes, regulating growth and metabolism by inhibiting the release of hormones such as *Gh* and *Igfs*. However, SST exerts specific regulatory effects on the secretion of *Gh* and *Igf* in different species. For example, catfish SS-22 does not inhibit the secretion of goldfish *Gh* [47], but it was able to inhibit the secretion of *Gh* from rat pituitary cells [48]. To explore the effect of SST on growth functions, the effects of four SST peptides on *Igf-1* and *Igf-2* expression in *S. argus* liver fragments were investigated. In *Oncorhynchus kisutch*, stimulation of SST expression in the hypothalamus impeded hepatic *Igf-1* expression [49,50]. In vitro culture of *O. mykiss* hepatocytes in the presence of SS-14 inhibited *Igf-1* expression [7]. It is worth noting that *SST1* in *S. argus* can significantly downregulate the expression of *Igf-1* mRNA in hepatocyte cultures in vitro, which was also observed in *O. mykiss* [7]. Furthermore, *SST3* and *SST6* reduced the expression of both *Igf-1* and *Igf-2* at 3 h of treatment at the concentrations of 1 and 10 μM. Taken together, these data suggest that SSTs have an inhibitory effect on the growth of *S. argus*. In contrast, SST5 had no discernable effect on *Igf-1* and *Igf-2* expression. Only *Igf-1* increased significantly at 6 h, suggesting SST5 may play a different role in mediating the growth regulation of *S. argus*. However, this requires additional study. These data indicated that SSTs (with the exception of SST5) can interact functionally with the growth axis. Further research is needed to determine growth factors related to SSTs.

## 5. Conclusions

In the present study, four somatostatin genes (*SST1*, *SST3*, *SST5*, and *SST6*) were identified and characterized in *S. argus*. Amino acid sequence alignments were performed to establish their structure. Phylogenetic and syntenic analyses clearly identified orthologous relationships between *S. argus* and other gnathostome SST genes, supporting the annotations. Based on the observation that specific SST genes were expressed in a tissue specific manner, it was speculated that SSTs perform different functions in different tissues. Liver explant cultures incubated in the presence of different SST peptides showed that all SSTs, with the exception of SST5, reduced the expression of *Igf-1* and *Igf-2*. This provides evidence that SSTs can act on the *Gh*-*Igf* pathways in *S. argus* to temper the growth of the animals. The data presented here provide a framework for future studies of the function of the SST genes in *S. argus*, and provides corroborating evidence regarding the evolution of SST genes in vertebrates.

## Figures and Tables

**Table 1:** The somatostatin family genes and their sequence characteristics in three teleost fish species and the spotted gar.

Species	Gene	Accession	Chr.	Position	Intron Number	Length (aa)	Mol. Wt. (KDa)	pI
*Scatophagus* *argus*	*SST1*	MN503273	linkage group 4	1,497,913–1,500,200	1	123	13.50858	5.927
	*SST3*	MN503274	linkage group 4	1,509,010–1,511,180	2	127	14.18519	7.087
	*SST5*	MN503275	linkage group 23	6,586,660–6,590,089	2	106	12.14712	5.137
	*SST6*	MN503272	linkage group 8	28,731,379–28,732,313	1	110	12.32939	7.054
*Danio rerio*	*SST1*	ENSDARG00000040799	15	36,156,986–36,158,851	1	114	12.44737	6.573
	*SST3*	ENSDARG00000033161	15	36,115,955–36,120,277	1	119	13.77676	5.743
	*SST5*	XM_001333046	7	19,971,621–19,975,163	1	107	12.39434	6.564
	*SST6*	ENSDARG00000031649	23	28,731,379–28,732,313	1	111	12.50558	6.636
*Ictalurus punctatus*	*SST1*	ENSIPUT00000002347.1	17	13,328,384–13,330,108	1	114	12.41834	6.556
	*SST3*	ENSIPUG00000001600	17	13,313,346–13,315,085	1	118	13.19919	6.906
	*SST5*	NC_030422.1	7	16,459,454–16,460,769	1	101	11.59656	7.196
	*SST6*	ENSIPUG00000009885	15	20,806,961–20,808,421	1	109	12.42848	5.960
*Lepisosteus oculatus*	*SST1*	ENSLOCG00000009439	LG14	19,404,762–19,407,399	1	114	12.47024	5.443
	*SST3*	ENSLOCG00000009445	LG14	19,441,987–19,443,409	1	116	13.18407	7.085
	*SST5*	XM_006627348	LG2	57,923,010–57,929,171	1	122	13.45959	9.055
	*SST6*	XP_006642047.1	LG25	11,726,683–11,728,536	1	109	12.13611	7.049

## Supplementary Materials

The following are available online at https://www.mdpi.com/2073-4425/11/2/194/s1, Figure S1. Nucleotide and deduced amino acid sequences of *S. argus* SSTs; Figure S2. Comparison of SST amino acid sequences between *S. argus* and other species; Figure S3. The cDNA structure of four SST genes from *S. argus*; Table S1. Primers targeting SST genes for cloning and detection of expression; Table S2. Sequence identity comparisons of *S. argus* SSTs with other vertebrates; Table S3. Genes information for syntenic analyses.
