# Supplementary material for: Identification, Expression, and Functions of the Somatostatin Gene Family in Spotted Scat (Scatophagus argus)
Source: Genes (Basel). 2020 Feb 12;11(2):194. doi: 10.3390/genes11020194 (PMC7073721; doi:10.3390/genes11020194)
Supplement: Supplementary file 1 [file genes-11-00194-s001.zip › Supplementary figure/Figure S1.docx]

*SST1*

c 1

acacacacacacacactcacggtgatcggtgacgtcagcggggtgtataagagccgcgcg 61

gacgggacagacccagaagatccgccgaccccgacagacagaccgactgactgacacgtg 121

atgaagatggtctcctcctcgcgcacccgctgcctcctcctgctcctcctctccctcacc 181

**M K M V S S S R T R C L L L L L L S L T** 20

gcctccatcagctgctcctccgccgcccagagagactccaaactccgcctgttgctgcac 241

**A S I S C S S A** A Q R D S K L R L L L H 40

cggaccccgctgctgggctccaaacaggacatgtctcggtcctccctggcagagctgctc 301

R T P L L G S K Q D M S R S S L A E L L 60

ctgtcagacctgctccaggtggagaacgaggctctggacgaggacgacttccccccggcc 361

L S D L L Q V E N E A L D E D D F P P A 80

gagggggaacccgaagacatccgcgtcgatctggaacgagccgccgccgccggcagcggg 421

E G E P E D I R V D L E R A A A A G S G 100

ccgctgctcgccccccgagagcggaaagccggctgcaagaacttcttctggaagacgttc 481

P L L A P R E R K  **A G C K N F F W K T F**  120

acttcctgctgagagcctcgtcatcttcgtcctcaccctgcgtcctcatcgcactccgta 541

**T S C** - 123

cagactgtcgatgattagtttgggtcaactgttttaatttttctgggctgattctttctg 601

aatgtaaacttgatgaaactatttttaatagttggtttgaataaaatctgtttgaga 658

*SST3*

gtttt 5

attgttaagactccatttaaagtcaataaggactaatggacgtttatttaacttgaatgt 65

cagcgttccttcatcaccctgcagcccgccagctgtcaatcaaacgcaccatgtcacatg 125

acccgcaggaggaggagcatcgggcttcaaaagagcagcaccctgtggaccaaaccagag 185

acaagagcagaaccagaaccaggacgaggaccagcagaagataccagaccagcagacagt 245

atgcagtgcgttcgttgtcctgccatcttggctcttgtggcgttggttctgtgcagtccc 305

**M Q C V R C P A I L A L V A L V L C S P** 20

ggtgtttcctctcagctcgacagagatcaggaccagaaccagaaccaggacctggacttg 365

**G V S S** Q L D R D Q D Q N Q N Q D L D L 40

gagctgcgtcaccaccggctgctgcaacgagctcgcagtgccggactcctgccacaggag 425

E L R H H R L L Q R A R S A G L L P Q E 60

tggagtaaacgtgcagtggaggacctgctggctcagatgtctctgcccgaagccgatggc 485

W S K R A V E D L L A Q M S L P E A D G 80

cagcgggaggctgaggttgtttccatggcaacaggaggaagggtgaacctggagaggtcc 545

Q R E A E V V S M A T G G R V N L E R S 100

gtggacgcccccaacaacctgccaccccgcgagcgcaaagctggctgcaagaacttctac 605

V D A P N N L P P R E R K **A G C K N F Y** 120

tggaagggcttcacttcctgttaaaggaatgcgccacccagccgaggccaacccttcacg 665

**W K G F T S C**  - 127

ggaccagctgaccaatcccagattacttggccttcacctgaatgactgtatggaccaatc 725

agcagctctctggcagcaacatacctgaataataaatgtaattatcaattaaagagagaa 785

atcag 790

*SST5*

gtcagtagttagtcactcctgtgtaatgttatgaatc 37

aagtgtttttggggaagggggttatggtactagttagtgatactggtctcacctgtcggc 97

actggactggtcacacagaaaggtgaggcgagtagcaacaccacactgaaacaaatacgt 157

atggtgcagctgcttctcgtggctttgttttcctctgtgctgctggtgcaggtcagcggt 217

**M V Q L L L V A L F S S V L L V Q V S G** 20 gtcccacgcagagacatgctgacagaaacactgagagcagacctggcaaatgacaaggat 277

V P R R D M L T E T L R A D L A N D K D 40 ctcgctcacttgctcctgctgaagttcgtgtctgaactgatggcggcgagaggagacgag 337

L A H L L L L K F V S E L M A A R G D E 60 atgctccccgagccggaggatgaggaggagcaggaggcaggagtcagggaggaggtgatg 397

M L P E P E D E E E Q E A G V R E E V M 80 cggcggcatcttgccctctcccaaagagagcgcaaggcgggctgccgcaacttcttctgg 457

R R H L A L S Q R E R K **A G C R N F F W** 100

aagacgttcacctcgtgctagcaccggaccagagctgctcaagctggagtgttgttgttg 517

**K T F T S C** - 106

tttttaattcctcttcttttcaagcataacttattccgtgtttaattactttgttggagc 577

tcaatcgattactgcaagatgccaaatgtgcaggtgttgtagcaattgtaagctgtgata 637

aggctgtatgttttgagatgggcagggatttacacagtgtccacaataaaaaagaaaatc 697

agtttaagagagtatgtttgttagttataataaatataaaaagaaacctca 748

*SST6*

gtttccaaatcttgtgacca 20

ccttgccgctaccaactgaactgcactttcttcaccactctcatctggatcctgggctgg 80

tctgagttgcacagactctctctctctctttctctccagcacactctcacttaagctctc 140

tttgagcctcctgctcctgtgactgtagctgtggtccgtcacctgcactcccgtcacagc 200

atgcagctcctggtggtgttagcagctctcatgggggttctgttcagcgttagagcagcc 260

**M Q L L V V L A A L M G V L F S V R A**  A 20

gccgtgcttcctgtggaggacaggagccccagccatgtgaacagggagctgaacaaagag 320

A V L P V E D R S P S H V N R E L N K E 40

cggaaggagctgatcctgaagctggtgtctggcttgttggacggagctctggacaccaac 380

R K E L I L K L V S G L L D G A L D T N 60

ctgttgccaggggaagcggcacccgtggatctcgaggagccgctggagtctcgtctggag 440

L L P G E A A P V D L E E P L E S R L E 80

gagagggctgtctacaacaggctatcactgcctcagcgtgaccgcaaagccccctgtaaa 500

E R A V Y N R L S L P Q R D R K **A P C K**  100

aacttcttctggaaaactttcacctcctgctaacagtgcccaaaaccacccggctctgcc 560

**N F F W K T F T S C**  - 110

tgccttctgtactccttccctcccagctccacatgaactgtagtagacctcagctgtaca 620

tatcatctacacctgtcagacatgcagcatcgatggacttcaccgagacagtgtgtttat 680

gtatcaatttatgtatacactgtatgtatttatgtatgtaacattttctttcagggtaaa 740

caataaagcatg 752

**Figure S1.** Nucleotide and deduced amino acid sequences of *Scatophagus argus* SSTs. Amino acid and nucleotide sequences are shown in uppercase and lowercase letters, respectively. Potential enzymatic cleavage sites are boxed. Signal peptides are in bold and somatostatin sequences are underlined in bold. Consensus polyadenylation signals are underlined.
