# Supplementary material for: Identification, Expression, and Functions of the Somatostatin Gene Family in Spotted Scat (Scatophagus argus)
Source: Genes (Basel). 2020 Feb 12;11(2):194. doi: 10.3390/genes11020194 (PMC7073721; doi:10.3390/genes11020194)
Supplement: Supplementary file 1 [file genes-11-00194-s001.zip › Supplementary figure/Figure S2.rtf]

SST1
H. sapiens      SST1 : --MLSCR-LQCALAAL-SIVLALGCVTGAPSDPRLRQFLQKSLAAAA--GKQELAKYFLAELLS-EPNQTENDALEPEDLSQ--AAEQDEMRLELQR---SANSNPAMAP :  98
M. fascicularis SST1 : --MLSCR-LQCALAAL-SIVLALGGVTGAPSDPRLRQFLQKSLAAAA--GKQELAKYFLAELLS-EPNQTENDALEPEDLSQ--AAEQDEMRLELQR---SANSNPAMAP :  98
M. musculus     SST1 : --MLSCR-LQCALAAL-CIVLALGGVTGAPSDPRLRQFLQKSLAAAT--GKQELAKYFLAELLS-EPNQTENDALEPEDLPQ--AAEQDEMRLELQR---SANSNPAMAP :  98
G. gallus       SST1 : --MLSCR-LQCALALL-SIALAVGTVSAAPSDPRLRQFLQKSLAAAA--GKQELAKYFLAELLS-EPSQTENEALESEDLSR--GAEQDEVRLELER---SANSNPALAP :  98 
A. carolinensis SST1 : --MLPCR-LPCALALL-SLALALGSGAAAPADPRLRQFLQKSLAAAAA-GKQELAKYFLAELLS-EPGQAENEALEPEELSRGGGAEQDEVRLELER---SASLNPALEP : 101 
L. chalumnae    SST1 : --MLSSR-LQCAFAFL-SLALVVSNISAAPSDLRLRQLLQKSLAAAA--GKQEVSKYSLAELLA-ELAQAENEALESDDFSK--TSDQDEVRLELER---SANGNPALAP :  98
P. annectens    SST1 : --MLSCR-FQCALVLL-SLAVVFSKVSAAPSDLRLRQLLQRSLAAAA--GKQELTKYSLAELLS-ELAQSENDALDSSDLSR--GADQDEVRLELDR---SANSSP-LAA :  97 
S. canicula     SST1 : --MWCSR-LQLSLALL-SIALAVLSVSSAPTDNRYREILQRAMAATGSGGKAELTKYSLAQLLS-ELENAENEALETDAMGG-----RNEVRLELER---SIN--PNLAN :  95
T. rubripes     SST1 : --MFSSN-SMCLLMLLLSLSASFSCSSAAHRDFKLPLLLQSNALMGN---KQEMTRSSLANLLLSDLLQVGNEAL-EENLALA-EGEPEEVHIDMER-AAAAGIGPFLTP : 101
O. niloticus    SST1 : --MNSSSRLRCLLLLLVSLTASISCTSAAQRDSKLRLLLHRTPLLGS---KQDMSRSSLAELLLSDLLQVENEALEEENFPLA-DGEPEDIRVDLER---AAGSGPLLAP : 101
D. rerio        SST1 : --MLSTR-IQCALALL-SLALAVSSVSAAPSDAKLRQLLQRSLLSPA--GKQELARYTLADLLS-DLVQAENEALEPEDLSR--GAEKDDVRLELER---AA--GPMLAP :  96
S. chuatsi      SST1 : --MVSSSRLRCLLLLLLSLTASISCSSAAQRDSKLRLLLHRTPLLGS---KQDMSRSSLAELLLTDLLQVENEALEEENFPLP-EGEPEDIHVDLERAAAAAGSGPLLAP : 104
A. sinensis     SST1 : --MLSSR-LQCALALL-SLALAVSSVSAATSEPRIRQLLQRALVASA--GKQDLLKYSLAELLS-ELAQSENDALASDELSR--AAEQNDVRLELER---SANGNPAMAP :  98
E. coioides     SST1 : MKMVSSSRLRCLLLLLLSLTASISCSSAAQRDSKLRLLLHRTPLLGS---KQDMSRASLAELLLSDLLQVENEALEEENFPLA-EGEPEDVHVDLER-AAAAGSGPLLAP : 105
S. argus        SST1 : MKMVSSSRTRCLLLLLLSLTASISCSSAAQRDSKLRLLLHRTPLLGS---KQDMSRSSLAELLLSDLLQVENEALDEDDFPPA-EGEPEDIRVDLER-AAAAGSGPLLAP : 105                             


H. sapiens      SST1 : RERKAGCKNFFWKTFTSC : 116
M. fascicularis SST1 : RERKAGCKNFFWKTFTSC : 116
M. musculus     SST1 : RERKAGCKNFFWKTFTSC : 116
G. gallus       SST1 : RERKAGCKNFFWKTFTSC : 116
A. carolinensis SST1 : RVRKAGCKNFFWKTFTSC : 119
L. chalumnae    SST1 : RERKAGCKNFFWKTFTSC : 116
P. annectens    SST1 : RERKAGCKNFFWKTFTSC : 115
S. canicula     SST1 : RERKAGCKNFFWKTFTSC : 113
T. rubripes     SST1 : RERKAGCKNFFWKTFTSC : 119
O. niloticus    SST1 : RERKAGCKNFFWKTFTSC : 119
D. rerio        SST1 : RERKAGCKNFFWKTFTSC : 114
S. chuatsi      SST1 : RERKAGCKNFFWKTFTSC : 122
A. sinensis     SST1 : RERKAGCKNFFWKTFTSC : 116
E. coioides     SST1 : RERKAGCKNFFWKTFTSC : 123
S. argus        SST1 : RERKAGCKNFFWKTFTSC : 123


SST3
S. canicula     SST3 : --MWCSRLQLSLALLSIALAVLSVSSAP-------------TDNRHREILQRALDQELLKDT------MMKLLPLVT---DVDTE-AGTLDVSNAET :  72
C. milii        SST3 : ---MQARCTLTLALLS--LAALGLSALP-------------THLRDQLVLQGSPAMELRDGV------VRELIQAVA---DAESK-PQSEEAAEDFA :  69
E. coioides     SST3 : MQ--CIRCPTILVLVALVLCSPGVFSQPDRDQDQYQNQDLDLELRHHRLLQRARSAGLLSQE-WSKRAVEDLLAQMS-LPEADTQ-REAEVVSMATG :  92
G. aculeatus    SST3 : MQ--YTRCPAILVLVGLVLCGPGVSSQRNRDQDQDQYQNQDLDPRPHLLLQRARGAGLLSQD-WSKRAVEDLLAQMS-LPEADSQ-LGAEVVSAATG :  92
L. americanus   SST3 : MQ--CIRCPAILALLALVLCGPSVSSQLDRE--QSDNQDLDLELRQHWLLERARSAGLLSQE-WSKRAVEELLAQMS-LPEADVQ-REAEDASMATG :  90
O. mykiss       SST3 : MKVCRIHCALALLGLALAICSQGAASQ------------PDLDLRSRRLLQRARAAAWPHRS----GVSERWRTFYPNCPCLRPR-KVKCPAGAKED :  80
O. latipes      SST3 : MQ--HARGPAFVVLAALALSSLGVSSQSDRDQDQFQNHDLDLELRHHRLLQRARSAGLLSQD-WSKRAIENMLARMP-DPEAEAE-REAEV------ :  86
T. rubripes     SST3 : MQ--RIHSSTILMLTTLVLCIQGVSSQPDRD--LNQNQDLEMEVRHHRLLQHVHGAGLLSQE-WSKRAVEDLIAQMS-MPEGTGQ-RDTEVVSMATG :  90
T. nigroviridis SST3 : MP--GVRSSTILVLTALVLSIQAVSSQPDRD--LDQNQDLDQEVRRHRLLQRAHGAGLLSQD-WSKRAVEDLIAQMS-LPEGTGQ-RDTEVVSMATG :  90
C. auratus      SST3 : MRLCELHCYLALLGLSLVLCGRCANSQL----------EPDLDFRHHRLLQRASATGQATRWDFTKRDVEKLLSLLS-IPEMEMREKGLSMAGESED :  86
C. commersonii  SST3 : MRLCELHCYLALLGLSLVLCDRGADSQL----------EPDMDFRHRRLLQRARAIGLATQD-WTKKDIEELLSQLS-LPEIEARENGVSTTGGNDD :  85
D. rerio        SST3 : MRLCELQCYLALLGLSLVLCGRSANSQL----------EPEMDFRHHRLLQRARAIGQAQEW--TKKDVEELLSLLS-MPEMQMRESDLSTTDENED :  84
S. chuatsi      SST3 : MQF-VVRCPAILALVALVLCGPGVSSQLDRDQDQYQNQDLDLELRHHRLLQRARSAGLLSQD-WSKRAVEDLLAQMF-LPEADTQ-REAEVVSMATG :  93
L. crocea       SST3 : MQ--CVRCPAIFALVALVLCGPGVSSQLDRDQDQSQNQDLDLELRHHRLLQRARSARLLSQD-WSKRAVQDLLAQMS-LPEAEAQ-QEAEVVSMATG :  92          
S. argus        SST3 : MQ--CVRCPAILALVALVLCSPGVSSQLDRDQDQNQNQDLDLELRHHRLLQRARSAGLLPQE-WSKRAVEDLLAQMS-LPEADGQ-REAEVVSMATG :  92


S. canicula     SST3 : SEENTTPMKFQHRQLGMRMRKN-CKNFFWKTYTLC : 106 
C. milii        SST3 : PDE-KAELRLA---LAPRWRKD-CKNFFWKTYTLC :  99
E. coioides     SST3 : GRMNLERSVDPPNNLPPRERKAGCKNFYWKGFTSC : 127
G. aculeatus    SST3 : GRLHLERSVDPPNNLPARERKAGCKNFYWKGFTSC : 127
L. americanus   SST3 : GRMNLERSVDSTNNLPPRERKAGCKNFYWKGFTSC : 125
O. mykiss       SST3 : LRVELERSVGNPNNLPPRERKAGCKNFYWKGFTSC : 115
O. latipes      SST3 : GRNNMERSVDA--NVPPRNRKDGCKNFYWKGFTSC : 119
T. rubripes     SST3 : GRMNLERSVDTTNNLSPPERKAGCKNFYWKGPTSC : 125
T. nigroviridis SST3 : GRMNLERSVDTTNNLPP--RKAGCKNFYWKGFTSC : 123
C. auratus      SST3 : LRLEQERSAESSNQLPTRVRKEGCKNFYWKGFTSC : 121
C. commersonii  SST3 : LHLELERSAENTNQLYPRERKAGCKNFYWKGFTSC : 120
D. rerio        SST3 : LRVELERSAESSNHIPARERKAGCKNFYWKGFTSC : 119
S. chuatsi      SST3 : GRMNLERSVDSPNSLPPRERKAGCKNFYWKGFTSC : 128 
L. crocea       SST3 : GRMNLERSVDAPNNLPPRERKAGCKNFYWKGFTSC : 127
S. argus        SST3 : GRVNLERSVDAPNNLPPRERKAGCKNFYWKGFTSC : 127             


SST5
O. mykiss       SST5 : MLCSQLQVLLVALSAS------------------------VFLARVSAAPHRDMLAELLRADSTKG---NEDLSRTLLLKMMSDLMSAAVGENEVLPDLEE--ALGVREE :  81
O. nerka        SST5 : MLCSQLQVLLVALSAS------------------------VFLARVSAAPHRDMLAELLRADTTKG---NEDLSRTLLLKMMSDLMSTAVGENEVLPDLEE--ALGVREE :  81
X. maculatus    SST5 : MLCSQMKVLVVALCSS------------------------MLALHVSSAPQMDELTETLQAELIS----DKDLTGLLLLRLMSELTASRGEEMLREQEEEEEDELGGRQR :  82
P. formosa      SST5 : MLCSQMKVLFVALCSS------------------------MLALHVSSAPQMDELTETLQAELIS----DKDLTGLLLLRFMSELMASRGEEMLREKQEEEEEELGGRQR :  82
O. niloticus    SST5 : --MLRSQVLLVALGLS------------------------VLLVRVSTAPYSDMLTETLRADLTN----DKDLTHWLLLKFMAELMAARGDETR-----------RGREEX :  69
O. latipes      SST5 : -------MLLAALGLS------------------------LLMACVSGAPQRNPLTHTKQLQLLKS---EVDPTRLLLMKLVLELVALRRQEMLQELEEE---ELGGRER :  73
T. rubripes     SST5 : -MLSQVQLLLVTSFSS------------------------MLLMEVSSAPRKHMPSELLPADPLK----DKDLAILILLRIISEVMAAEREMLLLPQEGEEEAGVREGEV :  81
T. nigroviridis SST5 : --MTRSQLLLAASFSF------------------------LLLMEAGGTPRKHK---FLPADSVK----DKDLALVTSLKFAFEGTAAQREMLLLLQEDEEDAGVREMMM :  77
A. mexicanus    SST5 : -MCSQLQVVLVALSV----------------------LV--LVSRVSAAPRGDVLTQLLQNEADTKEN--EDISRMLMLKLLSELEVAGEN---EVLS-----GADVR-N :  74
D. rerio        SST5 : -MFSRLQVVVVTLWVS------------------------LLLCRVSSAPRGDLLFQLLRSQVDPKENELQDLSRLLLLKQLSESVTPEEKDALDSID-----ELDVR-N :  79
I. punctatus    SST5 : -MSSQMQIVVLAVSL----------------------LV--LVSRVRTAPRTDLLAHMLQNEADGK----EDLSRILLLKLISELKIPDEN---DTLS-----DDDMELN :  73
P. promelas     SST5 : -MFSPLQMVVATLSLS------------------------LLLCSVSSAPGGDMLIQLLRSEVEPKEIELQDFFRLLLLKQLSESAAPEEKQPRESIDD----DPEVH-N :  80
P. nattereri    SST5 : -MNFEVKSSLLSRAAGRSASTPVIQTFEEFEYFKSICLLNHLITRVSAAPRGDILAQL---QADPKGN--EELSRMLILKLLSDLEIPGDN---EVLS-----GTDVR-S :  95
G. aculeatus    SST5 : -----MQVLLVALFSS------------------------VLLVQVSGAPRGDMPTQ--RGDPAD----NKDLAYSMLLKFVSELTAARGGETLLVPEQEEEEAEGGRQE :  75
S. argus        SST5 : ----MVQLLLVALFSS------------------------VLLVQVSGVPRRDMLTETLRADLAN----DKDLAHLLLLKFVSELMAARGDEMLPEPEDEEEQEAGVREE :  78
                                     

O. mykiss       SST5 : V--RQLPLSHRERKAGCRNFFWKTFTSC : 107
O. nerka        SST5 : VV-RQLPLSHRERKAGCRNFFWKTFTSC : 108
X. maculatus    SST5 : LMRRHIRFTHRERKAGCRNFFWKTFTSC : 110
P. formosa      SST5 : LMRRHIRFSHRERKAGCRNFFWKTFTSC : 110
O. niloticus    SST5 : LMRRHLSLSQRERKAGCRNFFWKTFTSC :  97
O. latipes      SST5 : LMKRHIRFTQRERKAGCRNFFWKTFTSC : 101
T. rubripes     SST5 : MMRRQVPFSQRDRKAGCRNFFWKTFTSC : 109
T. nigroviridis SST5 : MMRRQIPFSQRERKAGCRNFFWKSYTSC : 105
A. mexicanus    SST5 : DVVRQLPFSQRERKTGCRNFFWKTFTSC : 102
D. rerio        SST5 : EVVRQIPVSQRERKAGCRNFYWKTFTSC : 107
I. punctatus    SST5 : RILRHLPLTTRERKAGCRNFFWKTFTSC : 101
P. promelas     SST5 : EVVRQIPLSHRERKTGCRNFYWKTFTSC : 108
P. nattereri    SST5 : DMVRQLPFPQRERKTGCRNFFWKTFTSC : 123
G. aculeatus    SST5 : VMRRHLPLTQRERKAGCRNFFWKTFTSC : 103
S. argus        SST5 : VMRRHLALSQRERKAGCRNFFWKTFTSC : 106   


SST6
G. gallus       SST6 : MQLVASLVSVLLLVWSVR-ATALPGEERLALQNSREQTKLRRDALLKMLAGLLESSD---------VASPDLEE--EGKLEEERAALGRLAQ :  80
L. americanus   SST6 : MQLLTSLVSFLLVVWSVR-ATALPIEEKLMSHNNVELTKERKELMLKMIAGLLEGIDSPPMGGE--VGSMDLEEPVEPKL-EERAAFNRLPQ :  88
E. coioides     SST6 : MQLLVVLAALMGVLFSVRAAAVLPVEDRSSIHVNRELSKERKELILKLVSGLLDGALDTNMLPGE-AAPVDLEEPLESRL-EERAVYNRLS- :  89
G. aculeatus    SST6 : MQLLVVLATLMGVLFSVRAAAVLPMEDRSSINVNRELSKERKELILKLVSGLLDGAADANMLPGE-AAPVDFEEPLESRL-EERAVYNRLS- :  89
O. mykiss       SST6 : MQLLVILASLMGVLYSVRAAAVLPVEERSP-LLNRELSKERKELILKLVSGLLDGATDTNMLPGEGVSPVDLEEPLESRL-EERAAYNRLSQ :  90
O. latipes      SST6 : MQLLVVLAALMGVLFSVRAAAVLPVEDRSSIHVNRELSKERKELILKLVSGLLDGSLDTNMLPGE-AAPVDFEEPLESRL-EERAVYNRLS- :  89
T. rubripes     SST6 : MQLLVVLAALTGVLLSIRAAAVLPVEERSPVHLNRELSKERKELILKLVSGLLDGALDTNMLP---METVDLEEPLESRL-EERAVYNRLS- :  87
T. nigroviridis SST6 : MQLLVVLAALTGVLLSVRAAAVLPVEERIPLHLNRELSKERKELMLKLVSGLLDGALDTNMLP---MEAVDLEEPLESRL-EERAVYNRLS- :  87
C. auratus      SST6 : MQLLSSLVSLLLVLYSVRAAAVLPVEERNP-AQSRELSKERKELILKLISGLLDGVDNSVLDGEIAPVPFDAEEPLESRL-EERAVYNRLSQ :  90
D. rerio        SST6 : MQLLASLVSLLLVLYSVRAAAVLPLEERNP-AQSRELSKERKELILKLISGLLDGVDNSVLAGEIAPVPLDVEEPLESRL-EERAVYNRLSQ :  90
I. punctatus    SST6 : MQLLASLVSLLLVLYSVRAAVVLPAEERSP-AHTREPSKERKEMILKMVSSLLDGVDNNMLAGDLL--PLEEEEPIDSRL-EERAVYNRLAQ :  88
A. sinensis     SST6 : MQLRASLVSLMLVVYSLRVVAVLPGEERLSVHSNRELSKERKEGFLKLLSGLLDGVDSSVVLGED-VSPMDLEEPLDSRL-EERAVYNRLSQ :  90
S. argus        SST6 : MQLLVVLAALMGVLFSVRAAAVLPVEDRSPSHVNRELNKERKELILKLVSGLLDGALDTNLLPGE-AAPVDLEEPLESRL-EERAVYNRLS- :  89
 

G. gallus       SST6 : LSQRDRKAPCKNFFWKTFTSC : 101
L. americanus   SST6 : LPQRDRKAPCKNFFWKTFTSC : 109
E. coioides     SST6 : LPQRDRKAPCKNFFWKTFTSC : 110
G. aculeatus    SST6 : LPQRDRKAPCKNFFWKTFTSC : 110
O. mykiss       SST6 : LPQRDRKAPCKNFFWKTFTSC : 111
O. latipes      SST6 : LPQRDRKAPCKNFFWKTFTSC : 110
T. rubripes     SST6 : LPQRDRKAPCKNFFWKTFTSC : 108
T. nigroviridis SST6 : LPQRDRKAPCKNFFWKTFTSC : 108
C. auratus      SST6 : LPQRDRKAPCKNFFWKTFTSC : 111
D. rerio        SST6 : LPQRDRKAPCKNFFWKTFTSC : 111
I. punctatus    SST6 : LPQRDRKAPCKNFFWKTFTSC : 109
A. sinensis     SST6 : LPQRARKAPCKN--------- : 102
S. argus        SST6 : LPQRDRKAPCKNFFWKTFTSC : 110


Figure S2. Comparison of SST amino acid sequences between S. argus and other species. The S. argus SST potential cleavage site is indicated with a black arrow. The SS-14 of S. argus is underlined. Black shadow indicates the same amino acids.
