# Supplementary material for: Identification, Expression, and Functions of the Somatostatin Gene Family in Spotted Scat (Scatophagus argus)
Source: Genes (Basel). 2020 Feb 12;11(2):194. doi: 10.3390/genes11020194 (PMC7073721; doi:10.3390/genes11020194)
Supplement: Supplementary file 1 [file genes-11-00194-s001.zip › Supplementary figure/Figure S3.docx]

*SST1*

CACACACACACACACACTCACGGTGATCGGTGACGTCAGCGGGGTGTATAAGAGCCGCGCGGACGGGACAGACCCAGAAGATCCGCCGACCCCGACTGACAGACCGACTGACTGACACGTGATGAAGATGGTCTCCTCCTCGCGCACCCGCTGCCTCCTCCTGCTCCTCCTCTCCCTCACCGCCTCCATCAGCTGCTCCTCCGCCGCCCAGAGAGACTCCAAACTCCGCCTGTTGCTGCACCGGACCCCGCTGCTGGGCTCCAAACAGgtgggacagacggcagctcacacagacagacagacaggcagagacagtgagacagtgagacaggacattcctttggtgaaacttttcttcttctggtgttttgctctctgaaccttctcggtgctgatttctcactttgcacgttcatgtcaaagcgccgcggctcgttttgtctcgtctcacgccaaaactctcgcgcacgcgactttatttaaccgtcagacggacggacgacacgacgtcagaccggcacgcgtctccagtaacatccgacagaatctgagctgcactttgctcatcagaaaatatccgaagtgattcagagatttcatttcttcaaattcagccttcagttgttgtttaaaaatctgaaaatcatcaaaatctgaaattttcacttgagacgatcagcaggtcaacacagaacagaaatgaaaagatgagacagaagcgacgagaaccgctcaaccaaaacaaaaccgcaagtccttccgctcgctttcgtcagccaagatgctgacagacagacagacagacaggcaggcaggcgggcagacaggcaggcaggacgggcagacagacagagaggcagacagacagacaggacaggcaggcaggcaggcagacaggcagacaggcaggcaggacgggcagacaggcaggacgggcagacagacagacaggacaggcagacaggcaggcaggacgggcagacaggcaggaaggcaggcagacagacagacaggacaggcagacaggcaggcaggacgggcagacaggcaggaaggcagacagacagacagacagacagacaggcaggcaggtcagtgtgagacagatgagatgttgtctgtggctctctggactctgccggtgatgaacgtttgttttgttttaaacatcgtctggttaatcaatagatgagcggctgcgtttagttgaatttatttcctctttagtcttcagattcttgtcttcttacatgacagaacaggaagtgatttttttctttctgaagagcagaaaattaacccggcaaactgtcaaaacaaagtgtactaatttaaatccaaacataagaggcgcctgatcctcctgaaggaacccaaagcctttgccctgcgttgtgtctctcaggttagaagcctgacaggcaaaactcagggcgaacacaatgcaaacgcagcttttagtcgatgctctcctaaaagagtagaacacgatgatgcgtgatgtctgacagtagaacacacatctctcatgaatctgagataaaatgtgtaattcagttcgaactgagccgtctaatccggactgaggatcagctgctggttcgggttcggtaaggcaggggatcagctcagggcctgagctgaggacggatcaatgactgaacgtatcagtgaaaataaagctggtgtgaaactttatttgtgatgtgtggggcttgactgtccagagacgccgtttggctttagacgtcaggaagagtttctggactcacagcaacgccttgtggtcgcctctttgtagtccatcacaggtgacacgtggccagtccgtccgcaagcacagcagggccggaaacactcggaacgccgtcgtggtgccgccggggcagcacggagaaacaaacccgacgcctcacgttcactcacagaacatgtcacaatgatttgattctgttgtcacgctttgtaatcgtcctgccgtgtgttttcagGACATGTCTCGGTCCTCCCTGGCAGAGCTGCTCCTGTCAGACCTGCTCCAGGTGGAGAACGAGGCTCTGGACGAGGACGACTTCCCCCCGGCCGAGGGGGAACCCGAAGACATCCGCGTCGATCTGGAACGAGCCGCCGCCGCCGGCAGCGGGCCGCTGCTCGCCCCCCGAGAGCGGAAAGCCGGCTGCAAGAACTTCTTCTGGAAGACGTTCACTTCCTGCTGAGAGCCTCGTCATCTTCGTCCTCACCCTGCGTCCTCATCGCACTCCGTACAGACTGTCGATGATTAGTTTGGGTCAACTGTTTTAATTTTTCTGGGCTGATTCTTTCTGAATGTAAACTTGATGAAACTATTTTTAATAGTTGGTTTGAATAAAATCTGTTTGAGA

*SST3*

GTTTTATTGTTAAGACTCCATTTAAAGTCAATAAGGACTAATGGACGTTTATTTAACTTGAATGTCAGCGTTCCTTCATCACCCTGCAGCCCGCCAGCTGTCAATCAAACGCACCATGTCACATGACCCGCAGGAGGAGGAGCATCGGGCTTCAAAAGAGCAGCACCCTGTGGACCAAACCAGAGACAAGAGCAGAACCAGAACCAGGACGAGGACCAGCAGAAGATACCAGACCAGCAGACAGTaggagtttcccttttcgcttacgttttgttttctggtcatctgtcttttatactttattctgatgcgtttggcttcccttcctccttcagtgtatattttcttcttctcctcctgatatttaggtATGCAGTGCGTTCGTTGTCCTGCCATCTTGGCTCTTGTGGCGTTGGTTCTGTGCAGTCCCGGTGTTTCCTCTCAGCTCGACAGAGATCAGGACCAGAACCAGAACCAGGACCTGGACTTGGAGCTGCGTCACCACCGGCTGCTGCAACGAGCTCGCAGTGCCGGACTCCTGCCACAGgtgagagagccaacaaccaatcagaacgcgatattatagccagctctttggactgctattccaactgaaagtcttcattacaacatcacaaaatcccaaaatgtccccccagattcagtcagtatttggaaattcaactttaaagagttcagacgatcaatcactttgttaactggaaggtgcaaatccaattattcattattcagtcattactttgttttttttaactttaaagcagggctgctccacagaaaacctgttggtcacatatgacaattccaagatcttaatgctaaaacagttgcacaagtaaaacaaaaatcatatcatccaaaaagacttggagatgtcagtgaagtttccagctttaatggattcatgctgagaggaccaagactcgaaccctgaggtaccaaactgaccagaggataatttgactaaagcaagattgttgtgtttattcgtacaattcgtgtgtgaaatgcatcgttcatagatggttttatttcccacacgacgtcgctgcacagctgggtacttccatataagtaaaatgtaaaatgtaatgcactgcttcattttatgtgttctacgagttcagcatttgctttgtatgttaggaaagtaaaatttcttctctcagttgtactgcagcagaagtacagtttataaagttatttgtacttaagtgaaagtactcagttacaccccaccgttgatggactcgtattaaaaacatctgaatgtcatgaacctctaaatggtaatgaagccggtgaggctgagcgagcagatgagcagtgaggatgcacagccattgttgtttctgcttgatttggtgccaaatgtttatggaccccctgctgtgagccgcactgcagctcgtgaacatgcatgactggagactgtagggaacatgtcaccttaaagtgagggaaacaaaggcaagagcagagcttcatcagcacgtggattccagcttgaaatgcacatttgatgagtttttactttaaggactttatgaagtataaaatattttacggttcagactggactgacgtctgttgtgacgcttcacgaactcttctcaaatgtgaaaacaaatcactgatttccagGAGTGGAGTAAACGTGCAGTGGAGGACCTGCTGGCTCAGATGTCTCTGCCCGAAGCCGATGGCCAGCGGGAGGCTGAGGTTGTTTCCATGGCAACAGGAGGAAGGGTGAACCTGGAGAGGTCCGTGGACGCCCCCAACAACCTGCCACCCCGCGAGCGCAAAGCTGGCTGCAAGAACTTCTACTGGAAGGGCTTCACTTCCTGTTAAAGGAATGCGCCACCCAGGTACCCAGCTGTCTGCCCCACCCCCTTCGGTAGACGATTACCTGTTGAAGTCCCGCCCAGCTGCCTGACTACCTGTGTAAGCCCTGCTTGGGTAACTTGAGTTCTGTGTCTCACCTGTCCCTGTTGTTTTGTTCCCAGCCGAGGCCAACCCTTCACGGGACCAGCTGACCAATCCCAGATTACTTGGCCTTCACCTGAATGACTGTATGGACCAATCAGCAGCTCTCTGGCAGCAACATACCTGAATAATAAATGTAATTATCAATTAAAGAGAGAAATCAG

*SST5*

GTCAGTAGTTAGTCACTCCTGTGTAATGTTATGAATCAAGTGTTTTTGGGGAAGGGGGTTATGGTACTAGTTAGTGATACTGGTCTCACCTGTCGGCACTGGACTGGTCACACAGAAAGGTGAGGCGAGTAGCAACACCACACTGAAACAAATACGTATGgtaagtcttcctatgaggctaaatttaaaccactcttttacttttcactttataaactcacctacacaaggtatgggaaaatatcttcattttcaagagcaaatgctgactcaggtgactgaacgtgagtcgatttgagagctgttagccgtgtgtttaccttcgcaaatgtcagccattttgactgactcacctgttgagaagctaattagcacatacctccgttttatcgacttttaggggaatgacgtgtgggtataaacaacagtgcttttactctctgatgaacagcttataattaaaaataaagaaacacaaaactcagacatattcataatatttcatcagacattcaagtaaactgttgcgtttgaggctctcagtgaaaatgcgatcttacagcgacgcccagtggacaagtgtgctttgtcgcagcatgaggctagtattaataatatatttccacatataaatactcttactggatcattattactgatgccctcacgagtatactagcttaatgtaataattctactgagttatgtgcaaatcagcaggagggtctgctgcttgttagctctaattaaccctgacaaaccgctgacacaatggcaatattttataacgtagcagcaggggtgggatgctgtgagggtctgagctgtgctcactgcaaaaagtcagctgtcatgaacaagaaaataagtgcattatagccattatatttatatttatattaaaaccaatgcagatactaacagcaagtgcacttgtctatccatccatttattatctataaaaataaacaaaacaagttgcaaattacaaaaaacaaaaaacaagtgaatattgccgtgatttaagatatttcatcttgcgtagatttcggtttttgcagcgtagtcatcacactgttggacccctctccttcctccaggagacaggcggacgcctacagtcacgacatcctcactacatggaaaatgttccttaaagtcaagctgtgtgatgattaagtactgagtaatcagaatgcaagtcctgtggtggcccaaaaggtcagaggttgtcaaactgaggacgcatgtggagcagaatcaagtttaaacagctggactaatgtgggaataactaacggtgcttttgttttctggttagcacagtcgattcagacagcaaaacatgagacccatttttgaaagggaggcaatgaaaaaggtaactcgtcagtcaaaatatgatgaaatcttgttggcctttagttaaccatcaatagagtgagcaagattagtatgatttttgtaactattttctataatggccaagtaaaaattaaataatgagtgaaacaggttcctaagggacatatgatgcaaaaagaactgctggactggaggaacacctgaagttcagcatcaacgttaagagtactccagcattgcatttctatttgttccagtagcctcaagccactctttctcgctaaagattttgttttttcattttcaaacttgcagtctgcagctcaaacctaccctgccgatcaatcagactctctggagccacacaagcttttatataactttgttcccatacaaaaaacctgtaaacacactttgacctcctgggtggtcctgaaccactgaactaagttaaccgtgtggcttagtggagcccatgaccttcccactcttctgacatgatgttttgaagtggtcaaacaataaattattgccacattttgttttcagacaaaatgaaatggatacaaaacccaaccattatttgccttttgatcaaacacggactgtaaacacagatcaaaatcaagtgttctgtttgcagtctgacatttagatgttctctgcttttatcttccactgactcaaagtgagtggtggagtgtcaggctggtttgtccaagcagcctcattaacatttgagtcacattgtgtcattaacagcactgcacagcatgcaggcctcatgttgagtccaggacacctgcaaagccaatgcggctaatcatgactgacagaagcaaagttatgtgctaccattaattaattagactttattgttgtgttttgcatccaatattttatccttaaagttattacaacatgagcgttgatcaacccaaagtcagatcgcggaggttttgtttttgcggacgtcttacaggtgcagttttgtgcctgtgtgcctcatgtcgtcaccgcagacgatgaccttatcctgtccagtgacacttttgttcacaacctccaccacgcacatcctcattccctcaccaggattcttctccaccaatcacatccttttgtagacttgacgatgaggagggcttgatgatgaagaagcctctttattagctctcggcacatcctacgcaaacagtgataagtgatttggtgattggtcggattttgttatattttggactttggacagtgctgcacatcatctgtgttgaagagagaataaacagatactggagttacatccttgtgtttgaggtggagatgctttgctctcagGTGCAGCTGCTTCTCGTGGCTTTGTTTTCCTCTGTGCTGCTGGTGCAGGTCAGCGGTGTCCCACGCAGAGACATGCTGACAGAAACACTGAGAGCAGACCTGGCAAATGACAAGgtgagtgactcaaatatgtagtggagatttaattggccgagtgtgtgagcagggaagtgcctcagaggagtgtgttaaagctgtatgaagccttgtgtgaagtgctatttttttttttttttttgctgccctccttcagGATCTCGCTCACTTGCTCCTGCTGAAGTTCGTGTCTGAACTGATGGCGGCGAGAGGAGACGAGATGCTCCCCGAGCCGGAGGATGAGGAGGAGCAGGAGGCAGGAGTCAGGGAGGAGGTGATGCGGCGGCATCTTGCCCTCTCCCAAAGAGAGCGCAAGGCGGGCTGCCGCAACTTCTTCTGGAAGACGTTCACCTCGTGCTAGCACCGGACCAGAGCTGCTCAAGCTGGAGTGTTGTTGTTGTTTTTAATTCCTCTTCTTTTCAAGCATAACTTATTCCGTGTTTAATTACTTTGTTGGAGCTCAATCGATTACTGCAAGATGCCAAATGTGCAGGTGTTGTAGCAATTGTAAGCTGTGATAAGGCTGTATGTTTTGAGATGGGCAGGGATTTACACAGTGTCCACAATAAAAAAGAAAATCAGTTTAAGAGAGTATGTTTGTTAGTTATAATAAATATAAAAAGAAACCTCA

*SST6*

GTTTCCAAATCTTGTGACCACCTTGCTGCTACCAACTGAACTGCACTTTCTTCACCACTCTCATCTGGATCCTGGGCTGGTCTGAGTTGCACAGACTCTCTCTCTCTCTTTCTCTCCAGCACACTCTCACTTAAGCTCTCTTTGAGCCTCCTGCTCCTGTGACTGTAGCTGTGGTCCGTCACCTGCACTCCCGTCACAGCATGCAGCTCCTGGTGGTGTTAGCAGCTCTCATGGGGGTTCTGTTCAGCGTTAGAGCAGCCGCCGTGCTTCCTGTGGAGGACAGGAGCCCCAGCCATGTGAACAGGgtgaggagggtgatttttattgttcatatgtacatactgacatagcattttctcttaatctcagctgttattattcacagatttgtccgttatcctttctgaaaaactaaataattaggaagctgaaaacaacgtaaaatcaagtttgcttgtgaaatcaacaaagaagtgatattttttattcaccttttaaaggattttaaagatatatatatttttaaaatttattctgttagttcataccattttaaaatgggaaaagtaaaaaaataatgatgctggagacttgagtatcctgaaagacagctgcaagaagttgacttatcatattttccatcatctagtctctttactaatattttcttttcttattttcttggtcagGAGCTGAACAAAGAGCGGAAGGAGCTGATCCTGAAGCTGGTGTCTGGCTTGTTGGACGGAGCTCTGGACACCAACCTGTTGCCAGGGGAAGCGGCACCCGTGGATCTCGAGGAGCCACTGGAGTCTCGTCTGGAGGAGAGGGCTGTCTACAACAGGCTATCACTGCCTCAGCGTGACCGCAAAGCCCCCTGTAAAAACTTCTTCTGGAAAACTTTCACCTCCTGCTAACAGTGCCCAAAACCACCCGGCTCTGCCTGCCTTCTGTACTCCTTCCCTCCCAGCTCCACATGAACTGTAGTAGACCTCAGCTGTACATATCATCTACACCTGTCAGACATGCAGCATCGATGGACTTCACCGAGACAGTGTGTTTATGTATCAATTTATGTATACACTGTATGTATTTATGTATGTAACATTTTCTTTCAGGGTAAACAATAAAGCATG

**Figure S3.** The cDNA structure of four SST genes from *S. argus*. Red and blue uppercase letters represent untranslated region sequence and open reading frame sequence, respectively. Intron sequences are shown in lowercase letters. Promoters and terminators are boxed. Consensus polyadenylation signals are underlined.
