# Supplementary material for: Identification, Expression, and Functions of the Somatostatin Gene Family in Spotted Scat (Scatophagus argus)
Source: Genes (Basel). 2020 Feb 12;11(2):194. doi: 10.3390/genes11020194 (PMC7073721; doi:10.3390/genes11020194)
Supplement: Supplementary file 1 [file genes-11-00194-s001.zip › Supplementary figure/Table S1.docx]

**Table S1.** Primers targeting SST genes for cloning and detection of expression.

| **Name** | **Sequence (5' to 3')** | **Size (base pair)** |
| --- | --- | --- |
| *SST1*-F | AGACAGACCGACTGACTGACAC | 448bp |
| *SST1*-R | CTGTACGGAGTGCGATGAGGA |  |
| *SST2*-F | TGCCGCTACCAACTGAACTG | 635bp |
| *SST2*-R | GTCCATCGATGCTGCATGTC |  |
| *SST3*-F | CCCGCCAGCTGTCAATCAAA | 646bp |
| *SST3*-R | GAGAGCTGCTGATTGGTCCAT |  |
| *SST5*-F | TGAGGCGAGTAGCAACACC | 396bp |
| *SST5*-R | AACAACAACACTCCAGCTTGAG |  |
| *SST1*-RT-F | GAGAGACTCCAAACTCCGCC | 183bp |
| *SST1*-RT-R | AGATCGACGCGGATGTCTTC |  |
| *SST3*-RT-F | TGACTGTAGCTGTGGTCCGT | 213bp |
| *SST3*-RT-R | CCAGAGCTCCGTCCAACAAG |  |
| *SST5*-RT-F | TTCCTCTGTGCTGCTGGTG | 138bp |
| *SST5*-RT-R | GCCGCCATCAGTTCAGACA |  |
| *SST6*-RT-F | GCGTTCGTTGTCCTGCCATC | 283bp |
| *SST6*-RT-R | AGGTTCACCCTTCCTCCTGTTG |  |
| *β-actin*-F | GAGAGGTTCCGTTGCCCAGAG | 145bp |
| *β-actin*-R | CAGACAGCACAGTGTTGGCGT |  |
