# Supplementary material for: Identification, Expression, and Functions of the Somatostatin Gene Family in Spotted Scat (Scatophagus argus)
Source: Genes (Basel). 2020 Feb 12;11(2):194. doi: 10.3390/genes11020194 (PMC7073721; doi:10.3390/genes11020194)
Supplement: Supplementary file 1 [file genes-11-00194-s001.zip › Supplementary figure/Table S2.docx]

**Table S2.** Sequence identity comparisons of *Scatophagus argus* SSTs with other vertebrates.

| **Species** | **Gene** | **Identity** | **Accession** |
| --- | --- | --- | --- |
| *Homo sapiens* | *SST1* | 46.1% | BC032625 |
| *Macaca fascicularis* | *SST1* | 45.2% | XP_005545499 |
| *Mus musculus* | *SST1* | 45.2% | NP_033241.1 |
| *Gallus gallus* | *SST1* | 50.4% | CN210817.1 |
| *Acipenser sinensis*  *Carassius auratus*  *Danio rerio*  *Takifugu rubripes*  *Lophius americanus*  *Siniperca chuatsi*  *Epinephelus coioides*  *Homo sapiens*  *Mus musculus*  *Anolis carolinensis*  *Pelophylax ridibundus*  *Latimeria chalumnae*  *Danio rerio*  *Ictalurus punctatus*  *Lepisosteus oculatus*  *Epinephelus coioides*  *Gasterosteus aculeatus*  *Lophius americanus*  [[*Oncorhynchus mykiss*](http://www.ncbi.nlm.nih.gov/Taxonomy/Browser/wwwtax.cgi?name=Oncorhynchus+mykiss)](http://www.ncbi.nlm.nih.gov/Taxonomy/Browser/wwwtax.cgi?name=Oncorhynchus+mykiss)  [*Oryzias latipes*](http://www.ncbi.nlm.nih.gov/Taxonomy/Browser/wwwtax.cgi?name=Oryzias+latipes)  *Takifugu rubripes*  *Tetraodon nigroviridis*  *Carassius auratus*  *Catostomus commersonii*  *Danio rerio*  *Siniperca chuatsi*  *Larimichthys crocea*  *Danio rerio*  *Takifugu rubripes*  *Tetraodon nigroviridis*  *Gasterosteus aculeatus*  *Ictalurus punctatus*  *Oryzias latipes*  *Salmo salar*  [*Oncorhynchus mykiss*](http://www.ncbi.nlm.nih.gov/Taxonomy/Browser/wwwtax.cgi?name=Oncorhynchus+mykiss)  [*Oncorhynchus nerka*](http://www.ncbi.nlm.nih.gov/Taxonomy/Browser/wwwtax.cgi?name=Oncorhynchus+nerka)  *Xiphophorus maculatus*  *Poecilia formosa*  *Oreochromis niloticus*  *Oryzias latipes*  *Ictalurus punctatus*  *Danio rerio*  *Takifugu rubripes*  *Tetraodon nigroviridis*  *Astyanax mexicanus*  *Pimephales promelas*  *Pygocentrus nattereri*  *Gasterosteus aculeatus*  *Gallus gallus*  *Latimeria chalumnae*  *Epinephelus coioides*  *Gasterosteus aculeatus*  *Oncorhynchus mykiss*  *Oryzias latipes*  *Takifugu rubripes*  *Tetraodon nigroviridis*  *Carassius auratus*  *Danio rerio*  *Ictalurus punctatus*  [*Acipenser sinensis*](http://www.ncbi.nlm.nih.gov/Taxonomy/Browser/wwwtax.cgi?id=61970) | *SST1*  *SST1*  *SST1*  *SST1*  *SST1*  *SST1*  *SST1*  *SST2*  *SST2*  *SST2*  *SST2*  *SST2*  *SST2*  *SST2*  *SST2*  *SST3*  *SST3*  *SST3*  *SST3*  *SST3*  *SST3*  *SST3*  *SST3*  *SST3*  *SST3*  *SST3*  *SST3*  *SST4*  *SST4*  *SST4*  *SST4*  *SST4*  *SST4*  *SST4*  *SST5*  *SST5*  *SST5*  *SST5*  *SST5*  *SST5*  *SST5*  *SST5*  *SST5*  *SST5*  *SST5*  *SST5*  *SST5*  *SST5*  *SST6*  *SST6*  *SST6*  *SST6*  *SST6*  *SST6*  *SST6*  *SST6*  *SST6*  *SST6*  *SST6*  *SST6* | 45.7%  57.5%  60.2%  72.3%  84.3%  93.4%  93.5%  92.1%  79.5%  85.6%  49.1%  75.6%  72.8%  77.2%  47.4%  50.4%  48.3%  92.1%  91.3%  40.2%  59.2%  61.3%  63.2%  77.9%  57.0%  46.2%  48.5%  61.5%  54.5%  52.1%  41.0%  44.2%  71.3%  52.7%  61.0%  96.1%  90.2%  88.1%  94.6%  89.0%  89.1%  80.2%  77.0%  69.7%  67.3% | FJ792687.1  Q9YGH5.1  AAH76254.1  ENSTRUG00000010048  P01169.1  JN034584  AAU93565.1  AAB66895.1  AAD51127.1  XP_008119733.1  AAC60094.1  NW_005819697  XP_021325528.1  XP_017341959.1  NC_023203.1  AAU93566.1  KT235756  P01170.2  Q91194.1  AU168379.1  ENSTRUG00000025350.1  ENSTNIT00000001563.1  AAF15306.1  AAK97071.2  XP_694143.1  JN104627  XP_010747105.1  NP_571802.1  NC_018911.1  KT235753  KT235754  P01172.1  KT235752  CA039604.1  BX909872.3  EV380196.1  XP_005804860.3  XP_007551472.1  XP_005464163.1  XP_020567335.1  XP_017327476.1  XP_001333082.1  [XP_029696862.1](https://www.ncbi.nlm.nih.gov/protein/XP_029696862.1?report=genbank&log$=prottop&blast_rank=1&RID=MYYY57ZY014)  ALD51532.1  XP_007243346.1  DT105503.1  XP_017578562.1  [ALD51533.1](https://www.ncbi.nlm.nih.gov/protein/926659908)  DQ279789  XP_005994063.1  AAU93567.1  ENSGACG0000007077  CX153272.1  BJ885293.1  ENSTRUG00000012370  ENSTNIG00000015178  AAD09631.1  BG307388  [XP_017343581.1](https://www.ncbi.nlm.nih.gov/protein/XP_017343581.1?report=genbank&log$=prottop&blast_rank=1&RID=MYYPV800014)  FJ792688.1 |
